# Oncogenic BRAF Alterations and Their Role in Brain Tumors

**DOI:** 10.3390/cancers11060794

**Published:** 2019-06-08

**Authors:** Felix Behling, Jens Schittenhelm

**Affiliations:** 1 Department of Neurosurgery, Eberhard Karls University Tübingen, 72074 Tübingen, Germany; felix.behling@med.uni-tuebingen.de; 2 Department of Neuropathology, Comprehensive Cancer Center Tübingen-Stuttgart and Eberhard Karls University Tübingen, Abt. für Neuropathologie, Calwer Str.3, 72074 Tübingen, Germany

**Keywords:** BRAF V600E, KIAA1549-BRAF, MAPK, astrocytoma, glioblastoma

## Abstract

Alterations of the v-raf murine sarcoma viral oncogene homolog B (BRAF) have been extensively studied in several tumor entities and are known to drive cell growth in several tumor entities. Effective targeted therapies with mutation-specific small molecule inhibitors have been developed and established for metastasized malignant melanoma. The BRAF V600E mutation and KIAA1549-BRAF fusion are alterations found in several brain tumors and show a distinct prognostic impact in some entities. Besides the diagnostic significance for the classification of central nervous system tumors, these alterations present possible therapy targets that may be exploitable for oncological treatments, as it has been established for malignant melanomas. In this review the different central nervous system tumors harboring BRAF alterations are presented and the diagnostic significance, prognostic role, and therapeutic potential are discussed.

## 1. Introduction

Like many other neoplasms, brain tumors, especially the ones originating from glia of white and grey matter, can harbor v-raf murine sarcoma viral oncogene homolog B1 (BRAF) gene alterations. Early animal models with Ras-1 induced glioma formation and experimental blocking of the BRAF hotspot mutation in brain tumor cell cultures suggest that tumor growth is similarly regulated via mitogen-activated protein kinase/extracellular signal-regulated kinase (MAPK/ERK) as seen in non-CNS tumors [1,2]. Interestingly, BRAF activation in human neural stem and progenitor cells not only promotes tumor growth, but also subsequently causes oncogene-induced senescence in some low-grade brain tumors [3]. This may explain the relatively high frequency of BRAF mutant brain tumors associated with favorable outcome. Furthermore, BRAF gene alterations are also found in diffusely growing tumors associated with a poor prognosis in adults. Continuous signaling of MAPK maintains tumor growth, but BRAF activation alone is mostly not sufficient to drive their malignant behavior. By exploiting a nicely constructed Cre/lox animal model Robinson and colleagues were able to demonstrate that the combination of BRAF mutation with Akt activation or Ink4a/ARF loss is required to generate brain tumors with high-grade appearance [4]. This highlights the important role of BRAF alterations in oncogenic signaling in brain tumors. Accordingly, there is a high clinical relevance of testing brain tumors for the presence of BRAF mutations/fusions as targeted therapies for BRAF aberrations are available with the clinical introduction for metastatic malignant melanoma [5]. Despite limitations imposed by the blood–brain barrier and adaptive tumor resistance to the growth-inhibitory effects, the possible application of BRAF targeted therapy in CNS tumors grows continuously. In this review we outline the current knowledge of BRAF gene alterations for the common tumor entities in children and adults.

## 2. Pediatric Brain Tumors

**Figure 1:**
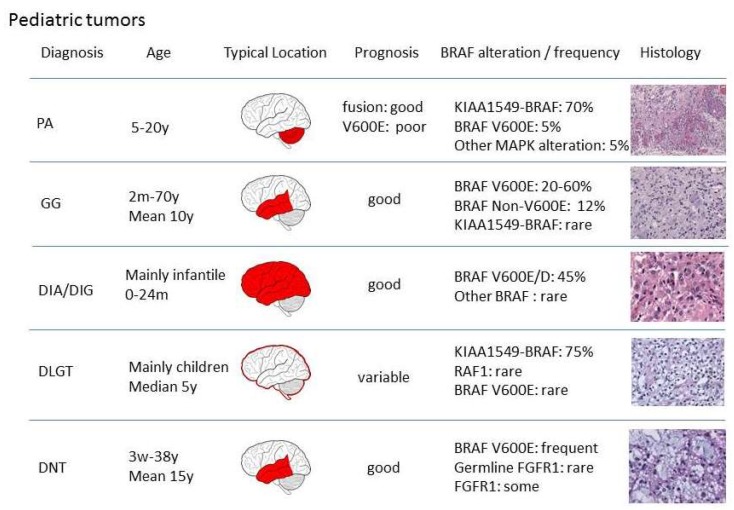
Overview of v-raf murine sarcoma viral oncogene homolog B (BRAF) alterations in pediatric brain tumors (PA: pilocytic astrocytoma, GG: ganglioglioma, DIA/DIG: desmoplastic infantile astrocytoma/ganglioglioma, DLGT: diffuse leptomeningeal glioneuronal tumor, DNT: dysembryoplastic neuroepithelial tumor.

Many pediatric low-grade gliomas (LGGs) and glial-neuronal tumors carry distinct molecular alterations with resultant aberrant intracellular signaling in the Ras-mitogen-activated protein kinase pathway. Among these, BRAF mutations and fusion transcripts are the most common genetic alterations (Figure 1). Especially the more common pilocytic astrocytoma (PA) and ganglioglioma (GG) are frequently associated with BRAF alterations. Less common tumor entities that harbor an oncogenic BRAF signaling are the epilepsy-associated dysembryoplastic neuroepithelial tumor (DNT) and the rare desmoplastic infantile gliomas (DIA/DIG). In 2016 the WHO classification of CNS tumors included another BRAF-associated glial tumor, the diffuse leptomeningeal glioneuronal tumor (DLGT), as a molecularly defined entity of BRAF-associated neoplasm. In this section we will discuss these tumors and the diagnostic and therapeutic implications of BRAF alterations in further detail below.

### 2.1. BRAF Fusions in Pilocytic Astrocytomas

Pilocytic astrocytoma is the most common astrocytic tumor in children and predominantly located in the infratentorial compartment [6]. When completely resected, this circumscribed and slow-growing tumor has a favorable outcome compared to other diffusely growing astrocytic tumors in the brain. However, such tumors may also grow in difficult locations such as optic pathways and the hypothalamus, making radical resection difficult. Almost all pilocytic astrocytomas are benign WHO grade I tumors and rarely show histological features of anaplasia. The most frequent genetic alteration in sporadic PA is a 2 Mb tandem duplication of chromosome 7q34 resulting in the transformation into a fusion gene involving the active BRAF kinase domain with replacement of the N-terminal domain by N-terminus of the KIAA1549 protein [7]. In rare cases, chromosome band 7q34 deletions resulting in similar KIAA1549:BRAF and FAM131B:BRAF fusions have been reported in these tumors [8]. Other recently reported fusions include GTF2I:BRAF and GIT2:BRAF [9]. The KIAA1549:BRAF fusions may involve at least nine different exon combinations, all of which contain an uncontrolled BRAF kinase domain resulting in a constitutively activated MAPK pathway [6,10]. The most common fusion is between KIAA1549 exon 16 and BRAF exon 9 (exon 16–exon 9) followed by KIAA1549:BRAF exon 15–exon 9 and KIAA1549:BRAF exon 16–exon 11. Non-BRAF fusions between SRGAP3 and RAF1 have also been found in rare cases [11]. As seen in KIAA1549:BRAF fused tumors, the activated RAF1 domain results in activation of the MAP kinase pathway [7]. The pilomyxoid astrocytoma variant is often associated with a more aggressive clinical course than classic PAs. Molecular examinations however show identical oncogenic KIAA1549:BRAF fusions associated with upregulation of some additional extracellular matrix proteins [12]. For this reason the grading in pilomyxoid astrocytomas was suspended until further clinical data is available. Approximately 15% of all patients with hereditary neurofibromatosis type 1 (NF1) eventually develop a pilocytic astrocytoma of the optic nerve [6]. The few remaining mutations identified in pilocytic astrocytoma involve BRAF V600E mutations, KRAS hotspot mutations, NTRK fusions, or FGFR1 duplications [13]. 

Up to 90% of all identified mutations in PA account for the MAPK pathway, thus indicating that pilocytic astrocytomas are likely a single pathway disease [6]. In these slowly growing tumors, the oncogenic BRAF activation or NF1 loss triggers oncogene-induced senescence through p16(INK4a) pathway induction following aberrant activation of the mitogen-activated protein kinase pathway [14]. Recent data suggests, that tumor associated micro-RNA is also involved in oncogenic senescence by directly targeting the MAPK/ERK and NF-κB signaling pathways [15]. Furthermore, the expression of the fusion product creates a supportive tumor microenvironment through NF-κB-mediated Ccl2 production and microglia recruitment [16]. Interestingly, the three most common BRAF fusions are not evenly distributed and are strongly associated with infratentorial tumor location and younger age of onset [17]. In contrast, the KIAA1549:BRAF exon15 to exon 9 fusions are more frequent in tumors located in the midline than in cerebellar tumors [18]. In general, somatic duplication of 7q34 is specific for pilocytic astrocytomas and molecular analysis of tumor samples for KIAA1549:BRAF fusions is diagnostic [19] in absence of co-occurring 1p deletions, which points to the differential diagnosis of a morphologically distinct diffuse leptomeningeal glioneuronal tumor [20]. In contrast, only 9% of BRAF V600E mutations are seen in pediatric pilocytic astrocytomas and these mutations overlap with the most likely differential diagnosis of ganglioglioma [21]. Presence of a BRAF-V600E mutation in PA is mutually exclusive with the presence of a KIAA1549-BRAF fusion. Currently, there is no difference in survival between tumors with BRAF duplication/fusion and wildtype, again which is in favor of the theory of PA being a single MAPK driven pathway disease. However, recent publication points to a potentially poor outcome in BRAF V600E mutated PA patients where the extent of resection and an additional CDKN2A deletion contributed independently [22].

Because of the high frequency of BRAF fusions, a patient-derived KIAA1549:BRAF-driven pediatric pilocytic astrocytoma model has been established for preclinical drug testing and has been used for selected MAPK and MEK inhibitor testing [23]. Treatment with first-line BRAF selective inhibitors such as vemurafenib or dabrafenib should be avoided in brain tumors with BRAF fusions. A phase II study using sorafenib in children with recurrent or progressive low-grade astrocytomas was terminated due to accelerated growth in pilocytic astrocytoma [24]. Because the KIAA1549-BRAF fusion signals as a dimer (in contrast to BRAF V600E monomeric activation of MAPK/ERK), one of the two molecules in the dimer is inhibited by targeted treatment, resulting in paradoxical activation of MAPK signaling by the other remaining molecule [25]. Therefore recent treatments have focused on downstream targets using MEK inhibitors in BRAF fused tumors. Consistent with their reduced capacity for paradoxical activation, second-generation inhibitors, such as PLX PB-3, inhibit both, the V600E BRAF monomer and the KIAA1549-BRAF fusion constitutive dimer equally well [26]. Partial responses have been reported for trametinib in progressively growing PA [27,28]. A phase I study using selumetinib in BRAF fused PA was recently completed and a 2-year progression free survival rate of 70% reported, suggesting good efficacy [29]. 

### 2.2. Gangliogliomas

Gangliogliomas are well differentiated, slowly growing mixed neuronal-glial tumors that are often located in the temporal lobe of children and young adults, typically causing early onset focal epilepsy. They have been reported in 15%–25% of patients undergoing surgery for chronic seizure control. It is still a matter of debate whether the seizures are caused by the tumor itself or are a result of molecularly induced alterations in the blood–brain barrier, associated inflammation, and disruption of glutamate homeostasis [30]. Studies with BRAF V600E mutant animal mouse models showed that they acquired intrinsic epileptogenic properties in neuronal lineage cells during brain development, whereas tumorigenic properties were attributed to high proliferation of glial lineage cells and responded to BRAF V600E inhibitor therapy [31]. As a WHO grade I tumor, the extent of surgical resection is the major factor affecting prognosis in ganglioglioma. A BRAF V600E mutation is the most common recurrent genetic alteration occurring in 20%–60% of all gangliogliomas [32]. The BRAF V600E mutation results in an activated protein that signals to MEK–ERK constitutively, stimulating cell proliferation and survival. There is a strong association of V600E-mutant tumors with age. The V600E mutation frequency is higher in young patients and less common in anaplastic ganglioglioma variants. The BRAF V600E mutation is mainly seen in mutant ganglion cells but may also be present in the glial component, suggesting that both components are derived from a common precursor with early mutation acquisition [33]. Other mutations detected in ganglioglioma by comprehensive molecular methods include a novel in-frame insertion at BRAF R506 in the β3-αC loop of the kinase domain, a CDC42BPB-BRAF fusion, KRAS Q61K mutations, an ERC2-RAF1 fusion, a germline NF1 mutation, and FGFR1/2 alterations [34]. The predicted biological consequence of the less common MAP kinase variants identified in this study is the activation of the same MEK-ERK signaling pathway as in BRAF V600E mutant gangliogliomas.

Previous studies indicated that gangliogliomas with BRAF V600E mutations have an increased risk for progression and recurrence [35], especially for tumors located in the brainstem, where BRAF V600E mutant gangliogliomas show a shorter progression free survival compared to wild type gangliogliomas [36,37]. Because BRAF V600E mutations have been reported in other low-grade neoplasms such as pilocytic astrocytoma, dysembryroplastic neuroepithelial tumors, and pleomorphic xanthoastrocytoma (discussed below), the presence of a V600E mutation in a low-grade glioma is not diagnostic. Gangliogliomas of the posterior fossa may show features of a pilocytic astrocytoma with focal gangliocytic differentiation. Some of these tumors have BRAF duplications and KIAA1549-BRAF fusions, suggesting that a minority of these tumors share a common molecular background with classical pilocytic astrocytomas [32,38]. This is further substantiated by methylation profiling studies in these tumors, forming a common subclass termed “hemispheric pilocytic astrocytoma and ganglioglioma” [39]. In the open-label, nonrandomized, multicohort VE-BASKET study, three anaplastic gangliogliomas and two pilocytic astrocytomas with BRAF V600E mutations were treated with vemurafenib twice daily and a partial response was observed in one patient with a pilocytic astrocytoma and one with an anaplastic ganglioglioma [40]. Sustained response was also observed in a reported ganglioglioma case with partial resection [41] and in a brainstem ganglioglioma treated with vemurafenib and vinblastine [42]. In a BRAF V600E mutated brainstem ganglioglioma, tumor regrowth was observed after withdrawal of vemurafenib [43]. Similar to observations made in melanoma, acquired resistance to prolonged vemurafenib treatment has been reported and persistent MAPK activation was suspected. In a case report of anaplastic gangliolioma, after resection of the relapsing tumor, a combined treatment consisting of vemurafenib and cobimetinib to overcome resistance was initiated and after 16 months of treatment, there was no evidence of recurrence [44]. A ganglioglioma patient who did not tolerate vemurafenib treatment due to cutaneous side effects was administered dabrafenib/trametinib instead and stable disease has been reported after 6 months follow-up [45]. Gangliogliomas are sensitive to pharmacologic and genetic autophagy inhibition and experimental data suggested that chloroquine can improve clinical efficacy of vemurafenib in a patient with a BRAFV600E tumor [46]. Of note, in a series of 54 pediatric midline gangliogliomas WHO grade I, five patients had co-occurring H3K27 and BRAF V600E mutations, showing that despite the presence of H3 K27M mutations, these cases should not be viewed as malignant midline gliomas and thus not treated as high grade tumors [47].

### 2.3. Desmoplastic Infantile Astrocytoma and Ganglioglioma

Desmoplastic infantile astrocytoma in its pure form and the desmoplastic infantile ganglioglioma [48]—when a variable neuronal component is present—are classically observed in infants. They are rare, often superficially located cystic lesions and can reach a large size resulting in increased head circumference and bulging fontanelles. Because the tumors are sharply demarcated, surgical resection is the treatment of choice and biological behavior usually corresponds to WHO grade I [49]. Somatic BRAF gene mutations were reported in approximately 45%, including BRAFV600E and BRAFV600D mutations. Other rare mutations involving BRAF are a reported FXR1-BRAF fusion [13] and a BRAF indel involving codons 600–604 [50]. Copy number analysis suggests that DIA and DIG represent a histologic spectrum of the same tumor type rather than two separate entities [51]. Rarely malignant transformation to glioblastoma has been observed in tumors acquiring additional TP53 mutations and some tumors may show leptomeningeal dissemination [49]. There is growing evidence that some of the non-BRAF altered DIA/DIGs are molecularly distinct tumors with ALK fusions [49]. The presence of BRAF V600E mutation is significantly associated with mTOR pathway activation and with a worse postoperative seizure outcome [52]. In such tumors targeted treatment of BRAF is a therapeutic option. A case report of a 3-year-old child with a nonresectable DIA harboring a BRAF V600E mutation that relapsed after conventional treatment was successfully treated twice with the BRAF V600E inhibitor vemurafenib, showing a fast response at the first treatment as well as at retreatment [53]. The previously reported case with a complex BRAF indel in codons 600–604 showed progressive leptomeningeal lesions besides standard chemotherapy. The treatment then was switched to BRAF-MEK inhibitors dabrafenib and trametinib at 8 months postoperatively and a marked decrease in residual tumor and leptomeningeal disease was observed 14 months after the initial surgery [50].

### 2.4. Diffuse Leptomeningeal Glioneuronal Tumor

Diffuse leptomeningeal glioneuronal tumor was recently introduced as a distinct entity in the 2016 WHO classification of CNS tumors. They show widespread diffuse leptomeningeal dissemination with tumor cells morphologically resembling oligodendrocytes. Due to the extensive leptomeningeal involvement, such lesions may be misdiagnosed as meningitis [54]. Although most tumors show a low-grade appearance, anaplastic features have been described and for this reason no WHO tumor grade is currently assigned to this entity. The most frequent genetic alteration is a KIAA1549-BRAF fusion reported in up to 75% of cases [20]. The major difference to KIAA1549-BRAF fused PAs are the concomitant 1p (or 1p/19q) deletion and the multifocality of DLGNTs. A rare case with a germline RAF1 mutation associated with pathological RAS pathway activation was described and a single case with BRAF V600 point mutation has been identified in DLGNT [55]. Recent data indicate that DLGNTs in children are distinct from those diagnosed in adults and that in up to 80% a MAPK/ERK pathway activation is present [56]. Despite the fact that up to 1/3 of children succumb to their disease, even with combined radiochemotherapy, there are no reports of DLGNTs receiving targeted treatment of the MAPK or mTOR signaling pathway [55]. 

### 2.5. Dysembryoplastic Neuroepithelial Tumor

The dysembryoplastic neuroepithelial tumor, often abbreviated DNT or DNET, is one major cause of early-onset temporal lobe epilepsy in children. The tumor is often located in the cortical layer and has a multinodular appearance with a diagnostic glioneuronal element in histology. Although the tumor is benign and graded as a WHO I lesion, recurrent seizures after long-term follow up have been described. As seen in other glioneuronal tumors, BRAF V600E mutations and FGFR1 alterations (including FGFR1 germline mutations) have been reported in up to 30%–80% of the tumors depending on the composition of the tumor cohort analyzed [57,58]. The wide dispersion in mutation frequencies and the absence of BRAF V600E mutations in later studies [59] is probably due to poor interobserver agreements regarding discrimination of DNT from other glioneuronal tumors such as ganglioglioma and probably improper classification of some tumors as DNET [60]. Combined molecular testing for BRAF V600E, KIAA-BRAF fusions and FGFR1 might be helpful in such cases [61]. Activation of mTOR and MAPK pathways suggest a role of altered signaling in DNT pathogenesis in up to 90% of these cases, particularly in the presence of a FGFR1 copy number gain [58,62]. To date, molecular testing of DNTs for FGFR1/BRAF is used as a diagnostic tool. However, targeted treatment of FGFR1-mutant DNT has not progressed to clinical trials yet. 

## 3. Adult Brain Tumors

**Figure 2:**
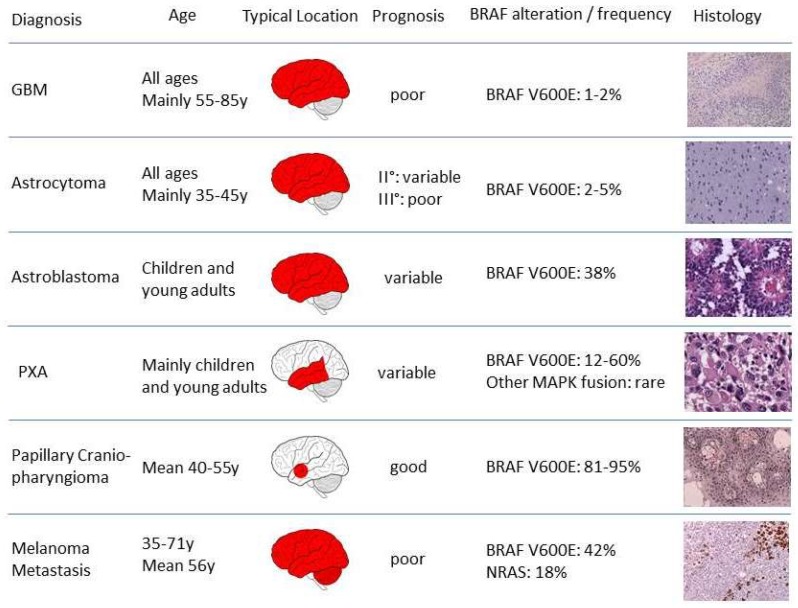
Distribution of BRAF alterations in adult brain tumors (GBM: glioblastoma, PXA: pleomorphic xanthoastrocytoma).

Shortly after the identification of BRAF as an important oncogene in cancer in 2002 [63], the first BRAF mutations in glioma have been described in 2004. They were found in a small subset of high-grade gliomas, associated with the V600 hotspot position and thus linked to a probable worse prognosis [64,65]. With the development of BRAF targeted inhibitors, the role of BRAF alterations in adult brain tumors was further investigated. A mutation specific monoclonal antibody (clone VE1) for rapid immunohistochemical detection of the BRAF V600E mutation was developed [66] and successfully applied and validated in central nervous system tumors. High throughput analyses with this antibody followed and revealed that BRAF V600E mutations are less frequent in adults (Figure 2), compared to pediatric brain tumors [32,67]. However, in light of the barely improved overall survival of patients suffering from high grade gliomas besides maximal multimodal treatment, BRAF mutated subgroups remain interesting entities that need to be further assessed for their accessibility to specific treatment modalities. In the following section we provide an overview of BRAF mutations in common adult brain tumors and their potential therapeutic implications.

### 3.1. Adult Glioblastomas

Glioblastoma is the most common primary brain tumor in adults and has a grim prognosis besides an aggressive multimodal treatment approach, consisting of maximal resection followed by radiochemotherapy [68]. Destructive invasive growth into the surrounding brain tissue leads to early tumor recurrence and a median overall survival of approximately 14 months [68]. Among the most common genetic aberrations, the MGMT promoter methylation is the only one that has a prognostic and predictive impact by causing an increased vulnerability of the tumor cells to alkylating agents and leading to a longer progression free and overall survival [69,70]. Currently, due to the prognostic potential, glioblastomas are separated by their IDH mutation status as primary (IDH-wildtype) and secondary (IDH-mutant) tumors [71]. 

BRAF V600E mutations are rarely found in adult gliomas with only 1% to 2 % mutated samples in glioblastomas and 2% to 5% in low grade adult gliomas [32,67]. Even though a clear prognostic difference could not be established yet, BRAF V600E mutant glioblastomas have some distinct histopathological and molecular features. BRAF mutations are in most instances mutually exclusive to canonical IDH mutations [61]. The few case reports in the literature with concomitant BRAF and IDH mutations were tumors without oncometabolic effects of bystanding non-canonical IDH mutations reported [72]. Furthermore, in some series, all BRAF V600E mutated glioblastomas showed distinct epithelioid features of the tumor cell morphology [67]. The tumor subgroup epithelioid glioblastoma (eGBM) was recently introduced into the WHO classification of tumors of the central nervous system as a rare variant of IDH-wildtype glioblastoma [71]. It was shown that BRAF V600E mutations are a common feature and seen in more than 50% of eGBM. They show typical radiographic features and are usually diagnosed at a younger age compared to classical GBM [73]. Even though the clinical behavior of mutated gliomas seems to be similar to wildtype tumors, screening for the BRAF V600E mutation is recommended for younger patients diagnosed with glioblastoma and in tumors with predominantly temporal lobe location, because mutation specific targeted treatment options exist [67] and encouraging case reports have been published. One case of eGBM with a BRAF V600E mutation was treated with vemurafenib and remained recurrence-free for 21 months [74]. Another eGBM case was treated with dabrafenib and was stable for 10 months [75]. Despite the uncertainties regarding the classification of eGBMs and its molecular relationship to other tumor entities, an existing BRAF V600E mutation can potentially be exposed to an established targeted treatment. This is of high clinical interest since gliomas tend to show recurrent growth even after aggressive treatment and are ultimately fatal. A few clinical cases of BRAF V600E mutant glioblastomas responding to small molecule inhibitor treatment with vemurafenib and dabrafenib have been published together with encouraging results. It is important to stress that the experience with these targeted treatment options in adults is limited compared to pediatric glioblastomas or relatively young adults [76,77,78,79]. Furthermore, first-generation BRAF inhibitors approved for adult melanoma have poor blood–brain penetrance and efficient response is only to be expected in tumors with markedly blood–brain barrier breakdown [80]. The investigation of BRAF inhibitors in glioma animal models showed a higher antitumor treatment efficacy in combination with MEK inhibition [81], EGFR inhibition [25] or concurrent radiotherapy [82] with longer animal survival compared to BRAF inhibition alone. These are encouraging results that also underline the importance of a multidirectional targeted treatment to overcome resistance mechanisms, which include feedback activation of mitogen-activated protein kinase or epidermal growth factor receptor signaling as seen in monotherapy with BRAF inhibitors. 

Gliosarcoma, a rare histopathological variant of IDH-wildtype GBM with mesenchymal and glial properties, is usually treated like GBM since controlled clinical trials are challenging due to its rarity. A recent large retrospective registry study revealed that the treatment response and prognostic factors are similar to GBM [83]. Initial studies suggested that BRAF mutations are absent in gliosarcomas. However, BRAF V600E mutations were first described in a few case reports [84,85] and later in a study applying next generation sequencing in 10 gliosarcomas, which revealed BRAF V600E mutations in 2 cases [86]. We recently analyzed a cohort of 75 gliosarcomas and only one case harbored a V600E mutation, making it comparably rare as in GBM [67]. The prognostic role of V600E-mutant gliosarcomas has not been studied yet. So far, no other BRAF mutations or fusions have been reported in gliosarcoma. Unfortunately, many clinical trials on GBM exclude gliosarcomas. The North American Brain Tumor Consortium study 05-02, investigating the efficacy of sorafenib, was open for gliosarcomas but did not include any of these tumors in the final cohort [87].

### 3.2. Diffuse Astrocytomas and Oligodendrogliomas

The rate of BRAF V600E mutations is quite low in diffuse gliomas ranging from 2% to 5% [32,67]. In a series of 106 diffusely growing cerebellar low grade astrocytomas BRAF V600E mutations and KIAA1549-BRAF fusions were observed in 19 cases and a predilection of BRAF aberrations to cerebellar localization of diffuse growing astrocytomas proclaimed. However, among the mutated cases were 8 pilocytic astrocytomas and 6 astrocytomas that were not further classifiable [88]. In contrast to the low frequency of BRAF V600E mutations, BRAF gains are common in low-grade diffuse gliomas with 1p/19q loss (up to 39%) [89]. BRAF testing is recommended in these cases, as upon progression to a higher-grade lesion and exhaustion of standard treatment, knowledge on BRAF mutation status may provide further treatment options. Accordingly, vemurafenib treatment in the VE-BASKET study was applied to anaplastic astrocytomas with confirmed objective response in 9% of malignant gliomas [40]. This suggests that BRAF monotherapy alone may not be sufficient in BRAF-V600E mutant astrocytomas.

### 3.3. Astroblastomas

Astroblastoma is a rare controversial tumor entity currently classified as *other astrocytic tumor* in the WHO classification of CNS tumors. It is usually seen in the hemispheres of young adults and has a characteristic histological appearance with prominent rosettes. A subset of these tumors shows recurrent rearrangements of the MN1 gene, located at 22q12.3-qter. Up to 38% of astroblastoma cases show BRAF V600E mutations [90]. Methylation analysis of these tumors revealed a close relationship to pleomorphic xanthoastrocytomas (PXA, see below) and indicated a less favorable clinical course compared to MN1-mutant astroblastomas [91]. Future studies are required to address the question whether astroblastomas belong to a morphological variant of PXA or remain a distinct tumor entity.

### 3.4. Pleomorphic Xanthoastrocytoma

Pleomorphic xanthoastrocytomas (PXA) are rare, often circumscribed glial tumors occurring in children and young adults. These slow growing tumors show regular recurrence besides surgical resection in 35.4% after 5 years [92]. Anaplastic features can be found in recurrent tumors, are rare in primary manifestations but are usually treated with additional radiation therapy and chemotherapy protocols [92,93]. Surprisingly, the mutation rate is highly dependent on the tumor grade. Up to 60% of PXAs WHO grade II harbor a BRAF V600E mutation and only 12% of anaplastic WHO grade III cases are mutated [93]. This phenomenon is suggestive of BRAF-induced oncogenic senescence in PXA as associated with BRAF-fused PA [3]. Another study showed a comparable mutation rate of 43% of V600E mutations in PXA [61]. It is still unclear if mutated PXAs have a different prognosis compared to wildtype cases after stratification for their WHO grade, but they show a predilection to temporal lobe location [33]. Besides the most common BRAF V600E mutation, a kinase activating fusion of NRF1-BRAF and ATG7-RAF1 was reported in anaplastic PXAs without BRAF V600E mutations [94]. 

The similar high frequency of BRAF V600E in epithelioid glioblastomas and PXAs suggests that both tumors may belong to one family with divergent morphologic features [95,96]. In this context one interesting clinical case should be noted that reported an eGBM recurrence after the initial diagnosis of a PXA [97]. Additionally, DNA methylation data has revealed that pediatric glioblastomas with PXA-like molecular features show a favorable biological behavior [98]. In the VE-BASKET study 7 BRAF-mutated PXAs were treated with vemurafenib and an objective response rate of 43% was achieved [40]. 

### 3.5. Papillary Craniopharyngioma 

Papillary craniopharyngioma is a histologically benign, epithelial cystic tumor occurring in the sellar region and deriving from embryonal remnants of the Rathke pouch. It is observed exclusively in adults. Most common symptoms are endocrinological dysfunction and vision disturbances. Because these tumors can invade adjacent brain structures, surgical resection can be difficult due to the risk of hypothalamic injury. Recurrence rates are high and incompletely resected tumors tend to show destructive growth into adjacent structures despite radiotherapy [99,100]. The vast majority of these tumors carry a BRAF V600E hotspot mutation making these tumors a distinct entity from the Wnt-associated adamantionous type craniopharyngioma seen in children [101]. Haston et al. could demonstrate that MAPK pathway activation regulates tumor proliferation in papillary craniopharyngiomas via the embryonic transcription factor Sox2 [102]. A few cases of BRAF V600E mutated papillary caraniopharyngiomas with good response to targeted treatment were reported [101,103,104]. These encouraging results have led to a phase II clinical trial that is currently recruiting adult patients with BRAF-mutated tumors for vemurafenib and cobimetinib treatment (NCT03224767).

## 4. Response to Mutation Specific Treatment in Brain Metastases

The first in-depth experiences with the treatment of cancer with BRAF V600E mutation specific targeted therapies were gained in the field of metastatic malignant melanoma. Frequently patients undergoing small molecule inhibitor treatment showed stabilization of tumor growth and improved overall survival [105,106,107]. However, the treatment seems to be less effective in the central nervous system where individuals often show new metastatic spread or growth during mutation specific treatment while other organ systems remain stable. It is assumed that the blood–brain barrier poses an obstacle that hinders the drug to establish efficacious levels in the tumor tissue. Control rates of cerebral metastatic disease are better for dabrafenib (31%) compared to vemurafenib (16%), presumably based on the better penetration of blood–brain barrier due to its smaller size and molecular structure [105,108,109,110]. 

While BRAF mutations are found in up to 66% of primary malignant melanomas [63], a study by Gugger et al. showed cerebral metastases harbor BRAF V600E mutations in 42% and NRAS mutations in 18% with a mean age of 56 years at diagnosis [111].

## 5. Outlook 

BRAF V600E mutation specific treatment has improved the overall survival for patients diagnosed with metastatic malignant melanoma. The transfer of this novel targeted treatment to other cancer types with BRAF alterations has been initiated for different brain tumors including papillary craniopharyngiomas of the sellar region and several clinical trials have been designed to provide evidence of antitumor activity beyond single case reports of treatment response. The VE-BASKET study, a non-randomized open label multicohort study of several BRAF V600E mutant central nervous system tumors, showed efficacy of vemurafenib treatment, especially in PXA [40]. However, more specific substances that are currently under development and the combination of established targeted treatment protocols have the potential to increase treatment efficacy. Currently several clinical trials are investigating targeted treatment in recurrent BRAF V600E-mutant gliomas of children and young adults (NCT01748149 and NCT02684058) as well as pediatric primary low-grade gliomas (NCT02684058). Since the efficacy of BRAF V600E mutation specific antibodies in the central nervous system is unclear, additional effort is put into the assessment of drug concentrations and treatment related ERK signaling pathway activity in resected recurrent tumor tissue and cerebrospinal fluid after prior dabrafenib and/or trametinib treatment (NCT03593993). 

Additionally, the development of new substances with higher potency and central nervous system penetration has improved the treatment response in metastasized melanoma with brain metastases. Even though there is still a lot of room for improvement, these findings are encouraging to hopefully provide improved substances and more efficacious combination therapies to treat central nervous system tumors with BRAF alterations in the future.

## 6. Conclusions

BRAF alterations can be found in several tumors of the central nervous system. In pediatric tumors they are often associated with oncogenic senescence while in other tumor entities they clearly contribute to tumor development and progression. The emerging importance of targeted therapy approaches in oncology together with the established role of BRAF mutation specific small molecule inhibitor treatment in metastasized melanoma have laid the foundation upon which the transfer to other tumor entities with BRAF alterations can be built. This encouraging development in the treatment of BRAF mutated tumor entities gives hope for the further advancement of personalized tumor treatment.
